# Cultural and linguistic responsiveness in long-term care: A scoping review protocol on programs for residents and staff

**DOI:** 10.1371/journal.pone.0343588

**Published:** 2026-02-26

**Authors:** Wenting Yan, Carmel L. Montgomery, Liz Dennett, Stephanie A. Chamberlain

**Affiliations:** 1 Faculty of Nursing, College of Health Sciences, University of Alberta, Edmonton, Alberta, Canada; 2 Geoffrey and Robyn Sperber Health Sciences Library, University of Alberta, Edmonton, Alberta, Canada; University of Saskatchewan, CANADA

## Abstract

**Background:**

The demographic landscape of Western countries has shifted to a more diverse one. Along with the trend of an aging population, a new problem has emerged, which is the increased linguistic diversity in the aging population in these countries. As people age and their care needs increase, they may not receive optimal care if they don’t speak the same language as their caregivers in long-term care facilities. Culturally and linguistically responsive long-term care services are important to ensure the best care for an aging population, but there is limited evidence in the literature on the scope and practice of these services. The objective of this scoping review is to map out the types of CLR programs in LTC settings and examine their core components and target populations.

**Methods:**

The Arksey and O’Malley framework, further developed by Levac and colleagues, will be employed in this scoping review. The research question was framed using the PCC framework. A comprehensive systematic search was developed with an experienced librarian and will be conducted in Scopus, CINAHL, Embase, Medline, PsycINFO, and Academic Search Complete. All primary study designs, including quantitative, qualitative, and mixed methods, will be included. Studies must focus on culturally and linguistically responsive care programs used or implemented in long-term care services. There will be no date or language limitations. Findings will be thematically synthesized to answer the research question.

**Conclusion:**

This review protocol provides a transparent process for how it will be conducted. We aim to contribute to a better understanding of what culturally and linguistically responsive care programs exist, how cultural and linguistic responsiveness is currently addressed across diverse care environments, and what gaps remain in long-term care.

## Introduction

### Demographic change and linguistic diversity in aging populations

Western countries are experiencing both growth in immigration and an aging population, presenting new demographic realities for health and social policy [1]. Population aging first started in high-income countries, and it affects both native-born citizens and immigrant populations who have settled there [2]. In Canada, among immigrants aged 65 and older who arrived between 2012 and 2016, more than 60% of them could not speak English or French [3]. In the United States, those over 65 contribute to 17% of the 43 million foreign-born individuals, and data from the Migration Data Hub show that approximately 16% of them do not speak English well [4,5]. Similar trends were found in Australia, where more than 37% of older adults were born overseas, and 10.6% of permanent migrants are not proficient in English [6,7]. As linguistically diverse older adults age, their health and care needs increase and they are more likely to need support from formal healthcare systems [8,9]. When healthcare is delivered in a language that is not the individual’s preferred or most often used language, the communication barrier may affect the quality of care they receive and therefore impact their health outcome [3,10–12]. Therefore, for this group of people, linguistically and culturally responsive care (CLR) becomes important, which is defined as care that recognizes, respects, and is responsive to individuals’ cultural and linguistic attributes in communication, care planning, and service delivery [13,14].

### Diverse linguistic needs in long-term care

For older adults, as their care needs increase, they move from receiving care in the community or at home to relying on more intensive and ongoing services. In this migration and aging context, the importance of CLR care becomes especially clear in settings where day-to-day communication is central to daily care and decision-making, such as long-term care (LTC).

LTC, including nursing homes or assisted living facilities, is a distinct sector that helps to provide comprehensive care when older adults’ needs exceed what can be met at home or in the community [15]. Therefore, LTC residents are often individuals with physical dependency, multiple chronic conditions, and a high prevalence of Alzheimer’s disease or other dementias [16,17]. As cognitive impairment progresses, many residents have language decline and even language reversal, in which the less ingrained second language is lost and the dominant original mother tongue re-emerges [18–20]. This change further exacerbates the language barriers at a time when care needs are most intensive. At the same time, cultural diversity within LTC is growing, creating challenges of acknowledging and accommodating residents’ cultural backgrounds and linguistic needs [21,22].

Therefore, high care dependency, residents’ demographic shifts (cultural and linguistic diversity), together with disease progression, create conditions in which mismatches between residents’ preferred language and the language used within care facilities are likely to occur. These mismatches form the basis of patient-facility language discordance in LTC settings. However, national data on language use in LTC is often incomplete or inconsistent across jurisdictions and countries. And therefore researchers have highlighted the importance of addressing language barriers and patient-facility/patient-provider language discordance to ensure equitable access to LTC services [23].

### Patient-facility language discordance and needs for culturally and linguistically responsive care

Most care provided in LTC settings is shaped by dominant local values and complexities [24]. Therefore, the language healthcare providers use at work for communication is typically the local official language. For individuals from minority language communities whose primary language differs from that spoken by the majority, this creates a patient-facility language discordance that can affect communication and trust between patient and provider, and ultimately affect health outcomes and the quality of care [10,25].

Delivering care in residents’ preferred language is important to creating a culturally and linguistically appropriate environment and ensuring high-quality LTC services [10]. Reducing language discordance not only improves the effectiveness of communication but also demonstrates respect for residents’ identities, culture, and values. Conversely, patient-facility Language discordance further emphasized the importance of developing CLR care programs as a critical component of patient-centered care for older LTC residents from official linguistic minority communities [17].

Such programs could take diverse forms, including culture-specific dietary services to respect food traditions and religious practices, recreational activities tailored to linguistic and cultural backgrounds, and more effective communication support such as bilingual staff and translator services. All of these are important components of care for people from culturally and linguistically diverse backgrounds to receive high-quality care [26].

### Rationale for this review

While the importance of CLR care has been increasingly recognized in recent years, existing research has largely focused on barriers to or experiences of accessing aged care services, and on specific older populations, such as those with dementia, or on general care practices within particular national settings. For example, studies have investigated the gaps or barriers in accessing aged care for culturally and linguistically diverse populations, with social exclusion as the consequence [8,26,27]. While these studies highlight the urgent need for CLR care, what remains unclear is what types of CLR programs exist in LTC settings, how these programs are implemented, what their core components such as approaches, strategies, and evaluation are, and which cultural or linguistic groups they are designed to support.

Such knowledge is important, as mapping out the global landscape can help us improve our understanding of what good practices are and what is still lacking, and inform the development of future initiatives or programs. Meanwhile, existing evidence on CLR care programs in LTC varies in scope, design, implementation approaches, and target populations, and is described across different contexts and types of literature. Given this heterogeneity, a scoping review is well-suited to provide a broad examination of available CLR care programs in LTC and to identify key gaps in the literature [28].

Therefore, this scoping review will identify and categorise CLR programs within long-term care. It aims to address a crucial knowledge gap by mapping the types of CLR programs in LTC settings and examining their core components and target populations. By examining these, we seek to inform future practice and program development to enhance the cultural relevance and inclusivity of care delivery in LTC.

## Methods

The scoping review has been registered on Open Science Framework (OSF) (https://doi.org/10.17605/OSF.IO/PN9CF). This scoping review will follow the Arksey and O’Malley methodological framework, which was further developed by Levac and colleagues [29,30]. The review procedure will follow the Preferred Reporting Items for Systematic Reviews and Meta-Analyses Extension for Scoping Reviews (PRISMA-ScR) statement [31]. Critical appraisal will not be conducted in this review, as the aim is to map and describe CLR care programs in LTC rather than to systematically assess their effectiveness. Therefore, six stages of this scoping review will be included as follows:

### (1) Identifying the research question

1. What programs have been implemented in LTC settings to enhance the cultural and linguistic responsiveness of care for minority residents
2. How are these CLR programs implemented in LTC settings, including their core components such as approaches, strategies, and evaluation methods?
3. Which cultural or linguistic groups have been the focus of these programs, and what approaches and strategies have been used to develop and deliver them?

### (2) Identifying relevant studies

Relevant published literature will be systematically searched in multiple databases (Scopus, CINAHL, Embase, Medline, PsycINFO, and Academic Search Complete) by an experienced health sciences librarian (LD) and with input from the other researchers on the team.

In this scoping review, a cultural or linguistic minority is defined as people who have immigrant, or ethnocultural, or linguistic minority backgrounds, based on the national or regional context. A cultural or linguistic minority will not be included in the search strategies because it is difficult to capture them all with specific terms. Instead, they will be manually screened and identified during the screening process using the definition, which will be operationalized during screening and data extraction through study-reported information such as migration background, ethnicity, race, language spoken or preferred by LTC residents, and the country or region in which the study was conducted. To avoid missing relevant studies, two reviewers will independently screen all records, with a third reviewer resolving any disagreements and making the final decision.

Therefore, the keywords for the final search strategies will include terms related to “long-term care”, “culturally responsive program”, and “linguistically responsive program”. An initial test search was done on Medline on June 20, 2025, combining the synonyms and MeSH subject headings of long-term care with synonyms of culturally or linguistically appropriate care. A sample set of results from this Medline search was reviewed to ensure the feasibility of the review and to identify any potential missed search terms. See S1 Appendix for the proposed Medline search strategy.

### (3) Study selection

#### Data management and eligibility criteria.

After retrieving relevant studies from all the databases, they will be exported into Covidence (www.covidence.org), a web-based tool for review management. Duplicates will also be automatically detected and removed by Covidence.

Studies will be included based on the following eligibility criteria (Table 1). For studies not in English, due to practical constraints, translations will be done using the paid subscription version of DeepL Pro (https://shorturl.at/zgHFs), a high-quality online translation tool, rather than by native speakers. We acknowledge that the use of machine translation may involve certain limitations and challenges, such as ambiguity, polysemy, and the influence of cultural and sociolinguistic factors on interpretation [32]. To ensure translation accuracy, key sections of each study including objectives, population characteristics, and intervention or program descriptions will be cross-checked by the review team, and translated findings will be interpreted cautiously.

**Table 1:** Eligibility criteria.

Category	Inclusion Criteria	Exclusion Criteria
**Population**	- Older adults residing in LTC settings, must be identified as having immigrant, ethnocultural, or linguistic minority backgrounds, based on national or regional context. Subgroups include older residents with dementia, physical disabilities, or mental health conditions. - Healthcare workers in LTC, including nurses, healthcare aides or assistants, and personal support workers, who are trained to provide CLR care. - Studies that include both residents and staff described above are eligible. - Studies that include both minority and non-minority residents will still be considered eligible if the delivered care program components related to cultural or linguistic minority groups are clearly reported or if the CLR program is primarily designed to address the needs of minority populations.	- Older adults residing in LTC settings without immigrant, ethnocultural, or linguistic minority backgrounds, relative to the country or context. - Studies in which minority groups cannot be identified will be excluded
**Concept**	- Studies involving programs, interventions, or initiatives that aim to enhance cultural and/or linguistic responsiveness in LTC, as long as these programs are targeted at or ultimately benefit residents from ethnocultural minority groups. - Must include recreational, social, or community-based programming designed to reflect residents’ cultural or linguistic identities. - May also include staff training or educational programs with clearly defined programmatic components (e.g., structured training sessions and ongoing educational initiatives) aimed to improve intercultural competence, language access, or diversity and inclusion in LTC.	- General programs with no cultural or linguistic adaptation. - Papers that only focus on residents’ or staff’s experience or challenges they may face without providing any information about the potential programs, interventions or initiatives will be excluded, as this review focuses on mapping CLR programs, rather than relying on perceptions and experience. - Programs, interventions, or initiatives that are culturally adapted but implemented in the local context for the general local population will be excluded.
**Context**	- Long-term care settings, nursing homes, supportive/assisted living, or residential aged care facilities.	- Non-LTC contexts, including acute hospitals, personal homes, independent living, or programs not connected to LTC
**Study Design**	- Peer-reviewed primary research studies, including qualitative, quantitative, or mixed-methods designs. - Grey literature, such as student dissertations and theses that include primary data collection	- Secondary research (e.g., systematic reviews, scoping reviews), commentaries, theoretical papers, blog posts, or editorials without primary data. - Conference abstracts
**Publication Year**	- No restrictions on publication year.	–
**Language**	- No restrictions on language	–

The screening will be divided into two stages and three reviewers will be involved in this process. Two reviewers (two trainees in the research term) will be involved in screening papers by reviewing both titles and abstracts independently. After this initial screening, the process will move to full-text readings for a more in-depth review by the same two reviewers. If any inconsistencies or disagreements occur during the screening at any stage, the third reviewer (WY) will be involved in solving the conflicts. Additionally, reference screening will be used to manually identify relevant literatures from citation lists of included studies.

After having all eligible studies determined, the PRISMA-ScR flow diagram will be used to report the flow of the screening process.

### (4) Charting the data

For data extraction, a data extraction form will be used to extract descriptive data from all studies meeting our inclusion criteria. Data extracted from each study will include Study Characteristic (record number in Covidence, author(s), year of publication, country where the study was conducted, language of publication, study type (quantitative, qualitative or mixed-methods), and type of LTC settings, etc.), Program Characterisitc (description of the program/intervention, program goals or objectives, core components and structure, delivery method, frequency and duration of intervention, etc), Target Population Characteristics (the demographic information of target population/residents or staff), Outcomes Measured/Reported (resident-level outcomes, staff-level outcomes and organizational outcomes), and Implementation Facilitators and Barriers.

Again, three reviewers will participate in this process. Firstly, two reviewers will independently extract the data, and any discrepancies will be resolved through discussion with the third reviewer. If there is any uncertainty about the included paper or missing data, the authors will be contacted for clarification.

### (5) Collating, summarising, and reporting the results

After extracting all the data from included studies, a thematic synthesis will be used to identify and generate themes to answer the review question [33]. Thematic analysis is a method used to identify, analyze, and report themes within data. It provides a detailed approach to organizing and describing data, facilitating a deeper interpretation of various aspects of the research [34]. Considering the heterogeneity the selected studies may have, thematic analysis is chosen for its structured approach to integrating diverse data types, while ensuring that the findings are grounded in the data and allowing for the analysis of differences between cases. [35]. Given the aim of mapping out this scoping review, the synthesis will be primarily descriptive. The unit of analysis for coding will be the extracted textual sentences or paragraphs describing program characteristics, target population characteristics, reported outcomes, and implementation facilitators and barriers from the included studies.

The analysis will follow Thomas and Harden’s stages of thematic synthesis: 1. Begin with free line-by-line coding of findings from included studies 2. Then, organize these free codes into related areas to generate “descriptive” themes with similar concepts 3. In the end, develop an “analytical” theme [33]. To ensure the quality of synthesis, two reviewers will be involved in this process. An initial subset (around 10%) of studies will be independently coded by both reviewers to develop themes. Any discrepancies in coding or theme development will be discussed and resolved through discussion. If agreement cannot be reached, a third reviewer will be consulted.

### (6) Consultation

We plan to conduct a stakeholder consultation with individuals with lived experience after we finish the synthesis within the research team. The consultation will be undertaken as stakeholder involvement to obtain confirmatory feedback on the synthesized findings. In total, three consultations will be conducted with participants representing key stakeholder perspectives, including one staff member working in LTC settings, and one older adult from a minority background residing in LTC and one family member or caregiver of a resident. Potential participants will be invited via email. A summary of the themes and findings from the previous steps will be shared for their review, and they will be invited to provide feedback on whether the themes and corresponding interpretations are clear, appropriate, and meaningful. The lead author will conduct the consultation in an informal way such as email communication or online meetings if needed, to seek participants’ feedback on the relevance and clarity of the synthesized findings in relation to the initial review questions. Therefore, the consultation will be an informal discussion and given this focused purpose, a small number of participants representing key stakeholder perspectives is considered sufficient for this stage.

Input from the consultation will be used to confirm the existing themes and to enrich the discussion of the final review paper. It will not be used to generate new themes or to modify the synthesis. For this reason, and because no identifiable personal information will be collected or analysed, ethical approval will not be sought for the consultation. Contributors’ involvement will be acknowledged in the final paper.

### Study status and timeline

At the time of submission (October 2025), the study is ongoing. The literature search has been completed, but record screening, data extraction, and data synthesis have not yet begun and no data extraction or synthesis has occurred. The protocol has not been modified in response to early findings. The projected timeline for the remaining stages is as follows: (1) Record screening: November 2025 – March 2026 (2) Data extraction: April 2026 – May 2026 (3) Data synthesis: May 2026 – June 2026 (4) Consultation: June 2026 – July 2026 (5) Final manuscript writing: July 2026 – August 2026. The submission of the final paper is expected by September 2026.

Since this study is a scoping review, no participant recruitment will be conducted. This timeline may be adjusted and any deviations from this timeline will be reported in the final publication

### Ethics and dissemination

Ethical approval is not necessary for this scoping review because no primary data will be generated. All data will be sourced from publicly available databases.

## Discussion

This scoping review protocol demonstrates a rigorous and transparent approach to mapping the evidence of CLR programs that have been implemented in LTC settings worldwide. The results of this review will be disseminated in an academic journal.

By identifying key program characteristics, target populations, and implementation strategies, the review will contribute to a better understanding of how cultural and linguistic responsiveness is currently addressed across diverse care environments and what gaps still exist. The findings will help inform and support the development of more inclusive care models, related policy and practice in the future, and highlight gaps where further research or innovation is needed to promote equity and person-centered care in LTC settings.

## Supporting information

S1 Appendix Search Strategies in All Datasets. (DOCX)

S2 Appendix PRISMA-P checklist. (DOCX)
