# Supplementary material for: Cultural and linguistic responsiveness in long-term care: A scoping review protocol on programs for residents and staff
Source: PLoS One. 2026 Feb 26;21(2):e0343588. doi: 10.1371/journal.pone.0343588 (PMC12944755; doi:10.1371/journal.pone.0343588)
Supplement: S1 Appendix — (DOCX) [file pone.0343588.s001.docx]

**S1 Appendix**

**Search Strategy**

**Ovid MEDLINE(R) ALL <1946 to June 20, 2025>**

1 residential facilities/ or assisted living facilities/ or homes for the aged/

2 exp Nursing Homes/

3 Long-Term Care/

4 ("long-term care" or "nursing home*" or "homes for the ag*" or "old age home*" or "care home*" or "residential care" or "residential facilit*" or "assisted living" or ((senior* or retirement) adj2 (home or facilit* or residence))).mp.

5 1 or 2 or 3 or 4

6 ("cultural* responsive*" or "cultural* relevan*" or "cultural* appropriate*" or "cultural* sensitiv*" or " linguistically responsive" or " linguistically relevant" or "linguistically appropriate").mp.

7 ((minority or "second language*" or bilingual* or multilingual* or multicultural or "foreign language" or "english as a second language" or "diverse cultur*" or "multi ethnic" or multiethnic*) adj8 (program* or activit* or entertainment or enrichment or visitor* or companion* or music* or recreation* or art* or exercis* or function* or educat* or staff or workforce or personnel or employee* or caregiver* or nurse* or worker* or aide$ or assistant* or training or "professional development" or intervention* or train* or workshop* or initiative* or event*)).mp.

8 ("language support" or "linguistic diversity" or "multilingual care" or "minority language" or "multicultural care" or "ethno-specific").mp.

9 6 or 7 or 8

10 5 and 9

**Embase (OVID interface)**

1 residential care/ or residential home/

2 assisted living facility/ or nursing home/ or residential home/

3 institutional care/ or home for the aged/

4 long term care/

5 ("long-term care" or "nursing home*" or "homes for the ag*" or "old age home*" or "care home*" or "residential care" or "residential facilit*" or "assisted living" or ((senior* or retirement) adj2 (home or facilit* or residence))).mp.

6 1 or 2 or 3 or 4 or 5

7 ("cultural* responsive*" or "cultural* relevan*" or "cultural* appropriate*" or "cultural* sensitiv*" or " linguistically responsive" or " linguistically relevant" or "linguistically appropriate").mp.

8 ((minority or "second language*" or bilingual* or multilingual* or multicultural or "foreign language" or "english as a second language" or "diverse cultur*" or "multi ethnic" or multiethnic*) adj8 (program* or activit* or entertainment or enrichment or visitor* or companion* or music* or recreation* or art* or exercis* or function* or educat* or staff or workforce or personnel or employee* or caregiver* or nurse* or worker* or aide$ or assistant* or training or "professional development" or intervention* or train* or workshop* or initiative* or event*)).mp.

9 ("language support" or "linguistic diversity" or "multilingual care" or "minority language" or "multicultural care" or "ethno-specific").mp.

10 7 or 8 or 9

11 6 and 10

12 limit 11 to conference abstracts

13 11 not 12

**APA PsycInfo (OVID interface)**

1 residential care institutions/ or exp nursing homes/ or assisted living/

2 ("long-term care" or "nursing home*" or "homes for the ag*" or "old age home*" or "care home*" or "residential care" or "residential facilit*" or "assisted living" or ((senior* or retirement) adj2 (home or facilit* or residence))).mp.

3 1 or 2

4 ("cultural* responsive*" or "cultural* relevan*" or "cultural* appropriate*" or "cultural* sensitiv*" or " linguistically responsive" or " linguistically relevant" or "linguistically appropriate").mp.

5 ((minority or "second language*" or bilingual* or multilingual* or multicultural or "foreign language" or "english as a second language" or "diverse cultur*" or "multi ethnic" or multiethnic*) adj8 (program* or activit* or entertainment or enrichment or visitor* or companion* or music* or recreation* or art* or exercis* or function* or educat* or staff or workforce or personnel or employee* or caregiver* or nurse* or worker* or aide$ or assistant* or training or "professional development" or intervention* or train* or workshop* or initiative* or event*)).mp.

6 ("language support" or "linguistic diversity" or "multilingual care" or "minority language" or "multicultural care" or "ethno-specific").mp.

7 4 or 5 or 6

8 3 and 7

**CINAHL Plus with Full Text (EBSCOhost interface)**

S1 (MH "Long Term Care") OR (MH "Nursing Home Patients") OR (MH "Residential Facilities+") OR (MH "Assisted Living") OR ("long-term care" or "nursing home*" or "homes for the ag*" or "old age home*" or "care home*" or "residential care" or "residential facilit*" or "assisted living" or ((senior* or retirement) N2 (home or facilit* or residence)))

S2   (MH "Cultural Sensitivity") OR (MH "Cultural Competence") OR (MH "Cultural Deprivation") OR (MH "Cultural Diversity+") OR (MH "Cultural Safety")  OR  ("cultural* responsive*" or "cultural* relevan*" or "cultural* appropriate*" or "cultural* sensitiv*" or " linguistically responsive" or "linguistically relevant" or "linguistically appropriate" OR ((minority or "second language*" or bilingual* or multilingual* or multicultural or "foreign language" or "english as a second language" or "diverse cultur*" or "multi ethnic" or multiethnic*) N8 (program* or activit* or entertainment or enrichment or visitor* or companion* or music* or recreation* or art* or exercis* or function* or educat* or staff or workforce or personnel or employee* or caregiver* or nurse* or worker* or aide$ or assistant* or training or "professional development" or intervention* or train* or workshop* or initiative* or event*)) OR "language support" or "linguistic diversity" or "multilingual care" or "minority language" or "multicultural care" or "ethno-specific")

S3  S1 AND S2

**(EBSCOhost interface)**

S1  (TI("long-term care" or "nursing home*" or "homes for the ag*" or "old age home*" or "care home*" or "residential care" or "residential facilit*" or "assisted living" or ((senior* or retirement) N2 (home or facilit* or residence))) OR AB("long-term care" or "nursing home*" or "homes for the ag*" or "old age home*" or "care home*" or "residential care" or "residential facilit*" or "assisted living" or ((senior* or retirement) N2 (home or facilit* or residence))) OR KW("long-term care" or "nursing home*" or "homes for the ag*" or "old age home*" or "care home*" or "residential care" or "residential facilit*" or "assisted living" or ((senior* or retirement) N2 (home or facilit* or residence))) OR SU("long-term care" or "nursing home*" or "homes for the ag*" or "old age home*" or "care home*" or "residential care" or "residential facilit*" or "assisted living" or ((senior* or retirement) N2 (home or facilit* or residence))))

S2  (TI("cultural* responsive*" or "cultural* relevan*" or "cultural* appropriate*" or "cultural* sensitiv*" or " linguistically responsive" or " linguistically relevant" or "linguistically appropriate" OR ((minority or "second language*" or bilingual* or multilingual* or multicultural or "foreign language" or "english as a second language" or "diverse cultur*" or "multi ethnic" or multiethnic*) N8 (program* or activit* or entertainment or enrichment or visitor* or companion* or music* or recreation* or art* or exercis* or function* or educat* or staff or workforce or personnel or employee* or caregiver* or nurse* or worker* or aide$ or assistant* or training or "professional development" or intervention* or train* or workshop* or initiative* or event*)) OR "language support" or "linguistic diversity" or "multilingual care" or "minority language" or "multicultural care" or "ethno-specific") OR AB("cultural* responsive*" or "cultural* relevan*" or "cultural* appropriate*" or "cultural* sensitiv*" or " linguistically responsive" or " linguistically relevant" or "linguistically appropriate" OR ((minority or "second language*" or bilingual* or multilingual* or multicultural or "foreign language" or "english as a second language" or "diverse cultur*" or "multi ethnic" or multiethnic*) N8 (program* or activit* or entertainment or enrichment or visitor* or companion* or music* or recreation* or art* or exercis* or function* or educat* or staff or workforce or personnel or employee* or caregiver* or nurse* or worker* or aide$ or assistant* or training or "professional development" or intervention* or train* or workshop* or initiative* or event*)) OR "language support" or "linguistic diversity" or "multilingual care" or "minority language" or "multicultural care" or "ethno-specific") OR KW("cultural* responsive*" or "cultural* relevan*" or "cultural* appropriate*" or "cultural* sensitiv*" or " linguistically responsive" or " linguistically relevant" or "linguistically appropriate" OR ((minority or "second language*" or bilingual* or multilingual* or multicultural or "foreign language" or "english as a second language" or "diverse cultur*" or "multi ethnic" or multiethnic*) N8 (program* or activit* or entertainment or enrichment or visitor* or companion* or music* or recreation* or art* or exercis* or function* or educat* or staff or workforce or personnel or employee* or caregiver* or nurse* or worker* or aide$ or assistant* or training or "professional development" or intervention* or train* or workshop* or initiative* or event*)) OR "language support" or "linguistic diversity" or "multilingual care" or "minority language" or "multicultural care" or "ethno-specific")

OR SU("cultural* responsive*" or "cultural* relevan*" or "cultural* appropriate*" or "cultural* sensitiv*" or " linguistically responsive" or " linguistically relevant" or "linguistically appropriate" OR ((minority or "second language*" or bilingual* or multilingual* or multicultural or "foreign language" or "english as a second language" or "diverse cultur*" or "multi ethnic" or multiethnic*) N8 (program* or activit* or entertainment or enrichment or visitor* or companion* or music* or recreation* or art* or exercis* or function* or educat* or staff or workforce or personnel or employee* or caregiver* or nurse* or worker* or aide$ or assistant* or training or "professional development" or intervention* or train* or workshop* or initiative* or event*)) OR "language support" or "linguistic diversity" or "multilingual care" or "minority language" or "multicultural care" or "ethno-specific"))

S3  S1 AND S2

**Scopus (Advanced )**

TITLE-ABS-KEY("long-term care" or "nursing home*" or "homes for the ag*" or "old age home*" or "care home*" or "residential care" or "residential facilit*" or "assisted living" or ((senior* or retirement) W/2 (home or facilit* or residence))) AND TITLE-ABS-KEY("cultural* responsive*" or "cultural* relevan*" or "cultural* appropriate*" or "cultural* sensitiv*" or " linguistically responsive" or " linguistically relevant" or "linguistically appropriate" OR ((minority or "second language*" or bilingual* or multilingual* or multicultural or "foreign language" or "english as a second language" or "diverse cultur*" or "multi ethnic" or multiethnic*) W/8 (program* or activit* or entertainment or enrichment or visitor* or companion* or music* or recreation* or art* or exercis* or function* or educat* or staff or workforce or personnel or employee* or caregiver* or nurse* or worker* or aide$ or assistant* or training or "professional development" or intervention* or train* or workshop* or initiative* or event*)) OR "language support" or "linguistic diversity" or "multilingual care" or "minority language" or "multicultural care" or "ethno-specific")
